# Loss of p27^Kip1^ promotes metaplasia in the pancreas *via* the regulation of Sox9 expression

**DOI:** 10.18632/oncotarget.5770

**Published:** 2015-09-21

**Authors:** Pauline Jeannot, Caroline Callot, Romain Baer, Nicolas Duquesnes, Carmen Guerra, Julie Guillermet-Guibert, Oriol Bachs, Arnaud Besson

**Affiliations:** ^1^ INSERM UMR1037, Cancer Research Center of Toulouse, Toulouse, France; ^2^ Université de Toulouse, Toulouse, France; ^3^ CNRS ERL5294, Toulouse, France; ^4^ Molecular Oncology, Centro Nacional de Investigaciones Oncológicas, Madrid, Spain; ^5^ Department of Cell Biology, Immunology and Neurosciences, University of Barcelona - IDIBAPS, Barcelona, Spain

**Keywords:** p27^Kip1^, cell cycle, CDK, pancreas

## Abstract

p27^Kip1^ (p27) is a negative regulator of proliferation and a tumor suppressor via the inhibition of cyclin-CDK activity in the nucleus. p27 is also involved in the regulation of other cellular processes, including transcription by acting as a transcriptional co-repressor. Loss of p27 expression is frequently observed in pancreatic adenocarcinomas in human and is associated with decreased patient survival. Similarly, in a mouse model of K-Ras-driven pancreatic cancer, loss of p27 accelerates tumor development and shortens survival, suggesting an important role for p27 in pancreatic tumorigenesis. Here, we sought to determine how p27 might contribute to early events leading to tumor development in the pancreas. We found that K-Ras activation in the pancreas causes p27 mislocalization at pre-neoplastic stages. Moreover, loss of p27 or expression of a mutant p27 that does not bind cyclin-CDKs causes the mislocalization of several acinar polarity markers associated with metaplasia and induces the nuclear expression of Sox9 and Pdx1 two transcription factors involved in acinar-to-ductal metaplasia. Finally, we found that p27 directly represses transcription of Sox9, but not that of Pdx1. Thus, our results suggest that K-Ras activation, the earliest known event in pancreatic carcinogenesis, may cause loss of nuclear p27 expression which results in derepression of Sox9, triggering reprogrammation of acinar cells and metaplasia.

## INTRODUCTION

During tumorigenesis, deregulated proliferation is one of the hallmarks of neoplastic changes due to constitutive activation of mitogenic signaling pathways, such as Ras, the most frequently mutated oncogene in human cancers, and to inactivation of the cells' growth inhibitory barriers like the p53 and Rb pathways [1, 2]. Progression through the cell division cycle is controlled by the sequential activation of cyclin-CDK complexes. One level of regulation of these complexes is provided through their interaction with CDK inhibitors (CKIs) [3].

The CKI p27^Kip1^ (p27) is an important component of the Rb pathway by inhibiting cyclin-CDK activity [3, 4]. Indeed, as a negative regulator of the cell cycle, p27 plays a critical role in establishing and maintaining quiescence, as illustrated by the phenotype of p27 knockout mice which are approximately 30% larger than their wild-type littermates due to hyperplasia in most tissues [5-7]. In keeping with this role, p27 protein levels are high in quiescent cells and decrease during G1 following mitogen stimulation [8, 9]. Although p27 levels are regulated at the transcriptional and translational levels, proteolytic degradation constitutes a major mechanism to control p27 expression [10, 11]. Several degradation pathways for p27 have been described and the best understood is instigated in late G1 and S phase by CyclinE-CDK2-mediated phosphorylation of p27 on Thr187, creating a recognition site for the Skp2-SCF E3 ubiquitin ligase, which ubiquitinates p27 and promotes its proteasomal degradation [12-16].

Consistent with its role in growth inhibition, p27 is also a tumor suppressor and mice lacking p27 are predisposed to both spontaneous and induced tumorigenesis [5-8, 17-19]. p27 is haplo-insufficient for tumor suppression and loss of one *Cdkn1b* allele is sufficient to promote tumor formation [8, 17-19]. Decreased expression of nuclear p27 is commonly observed in many types of cancers in human and is a significant prognostic marker [20, 21]. However, unlike canonical tumor suppressors, p27 mutations are rarely observed in cancer and p27 is preferentially inactivated either via increased proteolytic degradation or exclusion from the nucleus [20, 22-25]. In fact, cytoplasmic localization of p27 has been associated with poor prognosis in several types of malignancies in human suggesting that it could participate in the pathogenesis of the disease [4, 20, 24, 26-29]. In mice, animals expressing a mutant form of p27 that cannot bind to cyclin-CDK complexes (p27^CK−^) are more susceptible to both spontaneous and induced tumor formation compared to p27 knockout mice, suggesting that p27 can act as an oncogene *in vivo* [19, 30, 31]. Subcellular localization of p27 is primarily controlled via phosphorylation events, with Ser10 phosphorylation promoting nuclear export and Thr157 (absent in mice) and Thr198 causing the cytoplasmic retention of the protein [20]. Activation of several oncogenic pathways results in the localization of p27 in the cytoplasm, including Akt, S6K1, Pim, and Ras [8, 17, 19, 20, 24, 32-36].

Over the past years, p27 has emerged as a multifunctional protein involved in the control of different cellular processes independently of CDK regulation, including migration and invasion, apoptosis, autophagy, progenitor/stem cell fate and specification, cytokinesis and transcriptional regulation [4, 37-40]. For instance, cytosolic p27 regulates cell migration and invasion by preventing the activation of RhoA [33, 38, 41, 42]. In transcriptional control, p27 acts as a transcriptional co-repressor when bound to specific transcription factors such as E2F4-p130 and Ets1 by recruiting the co-repressors HDAC1 and mSin3A to the promoters [37]. Interestingly, p27 could regulate different subsets of genes either in a CDK-dependent or -independent manner [37, 43]. In this way, p27 was found to play an important role in the repression of Sox2 expression during stem cells differentiation [44].

Pancreatic ductal adenocarcinoma (PDAC) is a very aggressive type of cancer with a median survival of less than a year and a 5-year survival rate inferior to 5% [45]. PDAC is a prime example of the multistep progression in carcinogenesis, both at the morphological and genetic levels [46]. PDACs are thought to arise from cells undergoing acinar to ductal metaplasia (ADM), a process in which acinar cells transdifferentiate into ductal cells, and progressively transition to pancreatic intraepithelial neoplasias (PanINs) that evolve to ever more dysplastic stages to become PDACs [45-51]. Similarly, the mutation pattern follows a relatively conserved course: K-Ras activation is found in the earliest stages, with concomitant activation of EGFR signaling and as preneoplastic lesions evolve, they accumulate other mutations, mainly inactivation of tumor suppressors, such as p16^INK4A^, p53, SMAD4 and BRCA2 [45, 46, 48, 50]. Murine models expressing activated K-Ras remarkably reproduce the evolution of the pathology and different stages of the human disease [47-51]. Pancreatic inflammation - pancreatitis, plays a critical role in promoting the early changes leading to PDAC formation and several mouse PDAC models have confirmed this hypothesis *in vivo* [47, 48, 52, 53]. During ADM, acinar cells dedifferentiate and re-express markers of pancreatic ductal progenitors such as Pdx1, Sox9, Hes1 and Hnf1b [51, 52, 54]. In fact, Sox9 expression is induced by activated K-Ras in acinar cells before metaplastic changes occur and is required for PanIN formation in K-Ras^G12D^ mice by promoting acinar to ductal reprogramming [51].

Loss of p27 nuclear expression is a frequent occurrence, between 46-70%, in pancreatic adenocarcinomas and is associated with poor prognosis [55-58]. While a fraction of PDAC exhibit mislocalization of p27 in the cytoplasm [56], it is not associated with negative prognostic, unlike other types of cancers in which cytoplasmic p27 is a marker of decreased survival compared to complete loss of the protein [24, 27-29]. In addition, deletion of a single p27 allele was sufficient to promote tumorigenesis in a K-Ras^G12D^-driven PDAC murine model, which was further accelerated when both alleles were absent, indicating that p27 inactivation may contribute to the development of PDAC [59].

Herein, we explored the possible contribution of p27 loss in pre-neoplastic changes in the pancreas. We found that K-Ras activation in the pancreas causes a redistribution of p27 in the cytoplasm of acinar cells. We next analyzed pancreata from p27-null and p27^CK−^ mice and found that in both lines, there was a mislocalization of acinar polarity markers such as Integrin β1 and Munc18 and re-expression of ductal progenitor markers like Pdx1 and Sox9. Furthermore, we found that p27 directly participated in the repression of Sox9 expression. Thus, our data suggests that loss of nuclear expression of p27 following K-Ras activation may promote Sox9 expression and ductal reprogramming and ADM.

## RESULTS

### K-Ras activation in the pancreas causes p27 mislocalization

**Figure 1:**
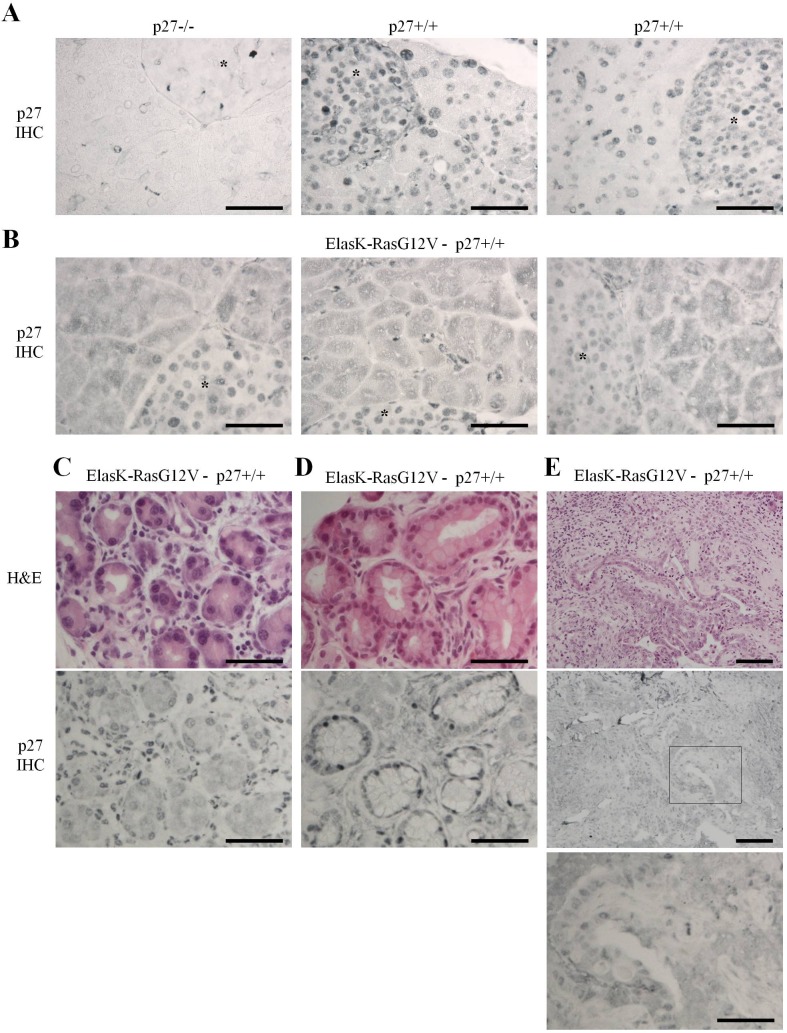
K-Ras activation causes loss of nuclear p27 localization Sections of paraffin embedded pancreas were stained for p27 or H&E. **A.**-**B.** Localization of p27 in normal pancreas of p27−/− (n = 3), p27+/+ (*n* = 3) and ElasK-Ras^G12V^ (*n* = 6) mice. Asterisks indicate β-islets. **C.**-**E.** H&E and p27 staining of consecutive sections in areas of ElasK-Ras^G12V^ pancreas showing either metaplasia **C.**, PanIN-1 **D.** or adenocarcinoma **E.** Scale bars in all images are 50 μm except in **E.** were the scale bars are 100 μm in the two top images.

Loss of p27 considerably shortens survival of mice in a K-Ras-driven PDAC model [59], indicating a prominent role for p27 in pancreatic cancer. To probe for p27's role in the pancreas, we first determined p27 levels and localization in pancreata from ElasK-Ras^G12V^ mice [47, 48]. Indeed, constitutive activation of N-Ras, H-Ras and K-Ras does not cause p27 degradation but was reported to induce mislocalization of the protein in the cytoplasm in various cell types and in the lung [8, 17, 19, 34, 35]. In absence of activated K-Ras, p27 is expressed exclusively in the nuclei of both acinar and β-islet cells when compared to background staining in p27−/− pancreas (Figure 1A). On the other hand, p27 localization was dramatically affected in ElasK-Ras^G12V^ pancreata: p27 was present in both cytoplasm and nuclei in most acinar cells while it remained completely nuclear in β-islet cells (Figure 1B), consistent with the Elastase promoter driving acinar cell-specific expression of K-Ras^G12V^ [47, 48]. In the different stages of pancreatic neoplastic progression, p27 was also seen in both cytoplasm and nucleus in areas of ADM (Figure 1C), PanINs (Figure 1D) and adenocarcinomas (Figure 1E). Thus, K-Ras activation causes at least partial loss of nuclear p27 in the pancreas, even in normal areas of the pancreas, before acinar to ductal metaplasia, suggesting that p27 inactivation may occur very early during pancreatic carcinogenesis.

### Loss of p27 affects acinar cell polarity

**Figure 2:**
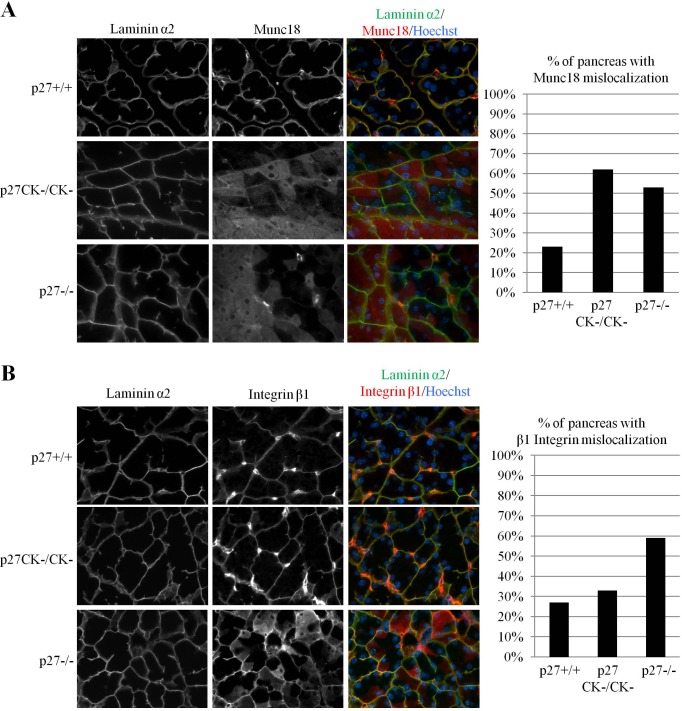
Mislocalization of integrinβ1 and Munc18 in acinar cells of p27^CK−^ and p27−/− pancreas **A.**-**B.** Sections of paraffin-embedded pancreas from p27+/+, p27−/−, and p27^CK−/CK−^ mice were stained for the basal markers laminin α2 and Munc18 **A.** or laminin α2 and Integrinβ1 **B.** DNA was stained with Hoechst. All images were acquired using a 60x objective. In **A.** the graph displays the percentage of p27+/+ (*n* = 12), p27^CK−/CK−^ (*n* = 13) and p27−/− (*n* = 12) mice with milocalized Munc18. In **B.**, the graph displays the percentage of p27+/+ (*n* = 11), p27CK−/CK− (n=12) and p27−/− (*n* = 17) mice with mislocalized Integrinβ1. Mice were considered positive for Integrinβ1or Munc18 mislocalization when several areas with non-basal staining were observed.

To determine if loss of p27 function may contribute to early events leading to ADM, we monitored several known markers of acinar polarity and ADM in the pancreas of p27^+/+^, p27^−/−^ and p27^CK−^ mice. Pancreatic acini are highly polarized structures and their architecture is completely remodeled during ADM or pancreatitis. For instance, Munc18 is located on baso-lateral membranes and acts as an inhibitor of SNARE-mediated membrane fusion to direct exocytosis to the apical membranes, preventing basal release of digestive enzymes [60-62]. During pancreatitis, localization of Munc18 to basal membranes is lost [60-62]. Munc18 immunostaining revealed that in p27-null mice, Munc18 basal localization was much more frequently lost than in p27^+/+^ pancreata (Figure 2A). Interestingly, Munc18 mislocalization was also observed in p27^CK−^ pancreata suggesting that the phenotype is a consequence of the loss of p27-mediated CDK inhibition (Figure 2A). On the other hand, we never saw alterations in the localization of the basement membrane component Laminin α2 (Figure 2).

Similarly, Integrin β1 is important for the maintenance of acini structure and pancreas-specific knockout of the protein leads to progressive organ degeneration similar to pancreatitis [62]. Integrin β1 immunostaining also showed loss of basal localization in a majority of p27-null mice (Figure 2B). However, to our surprise this phenotype was not as frequently observed in p27^CK−^ pancreata, or with only mild delocalization of Integrin β1 which was not counted as positive in our quantification (Figure 2B). Interestingly, Ki67 immunostaining did not show any significant changes in cell proliferation in the pancreas of adult p27^+/+^, p27^−/−^ and p27^CK−^ mice (data not shown). Thus, our data indicate that loss of p27 promotes the mislocalization of acinar polarity markers in the pancreas.

### Loss of p27 causes re-expression of pancreatic ductal progenitor markers

**Figure 3:**
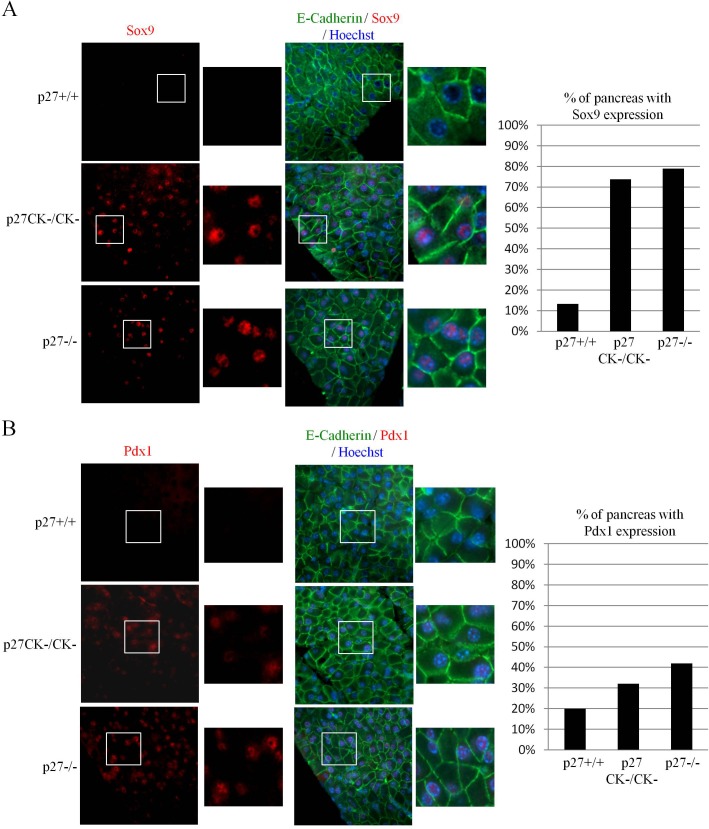
Expression of Sox9 and Pdx1 in acinar cells of p27^CK−^ and p27−/− pancreas **A.**-**B.** Sections of paraffin-embedded pancreas from p27+/+, p27−/−, and p27^CK−/CK−^ mice were stained for E-Cadherin and Sox9 **A.** or E-Cadherin and Pdx1 **B.** DNA was stained with Hoechst. All images were acquired using a 60x objective. The graphs display the percentage of p27+/+ (*n* = 15), p27^CK−/CK−^ (*n* = 19) and p27−/− (*n* = 19) pancreas with Sox9 expression **A.** and Pdx1 expression **B.** Mice were considered positive for Sox9 when more than a half of the pancreas expressed Sox9. Mice were considered positive for Pdx1 when more than a quarter of the pancreas expressed Pdx1.

ADM is a transdifferentiation process thought to precede PanIN formation in which acinar cells change their morphology to acquire ductal characteristics. This reprogrammation is accompanied by the re-expression of ductal progenitor markers such as Pdx1, Sox9, Hes1 and Hnf1b [51, 52, 54]. Sox9 levels were monitored by immunostaining and we found elevated expression in large areas of most pancreata of both p27−/− and p27^CK−^ animals (Figure 3A). Thus our results suggest that loss of p27-mediated CDK inhibition is sufficient to allow the re-expression of Sox9. Similar results were obtained when Pdx1 expression was investigated, albeit at a much lower incidence (Figure 3B). Pdx1 is abundantly expressed in β-islet cells and we did not observe any differences in Pdx1 levels in β-islets of p27^+/+^, p27^−/−^ and p27^CK−^ pancreata (Supplemental Figure 1). Altogether, our data suggest that loss of p27 promotes changes in polarity marker localization and pancreatic ductal progenitor markers typically observed in pre-metaplastic states and more specifically that the loss of p27-mediated CDK regulation is sufficient to promote these changes.

### p27 directly participates in the repression of Sox9 transcription

**Figure 4:**
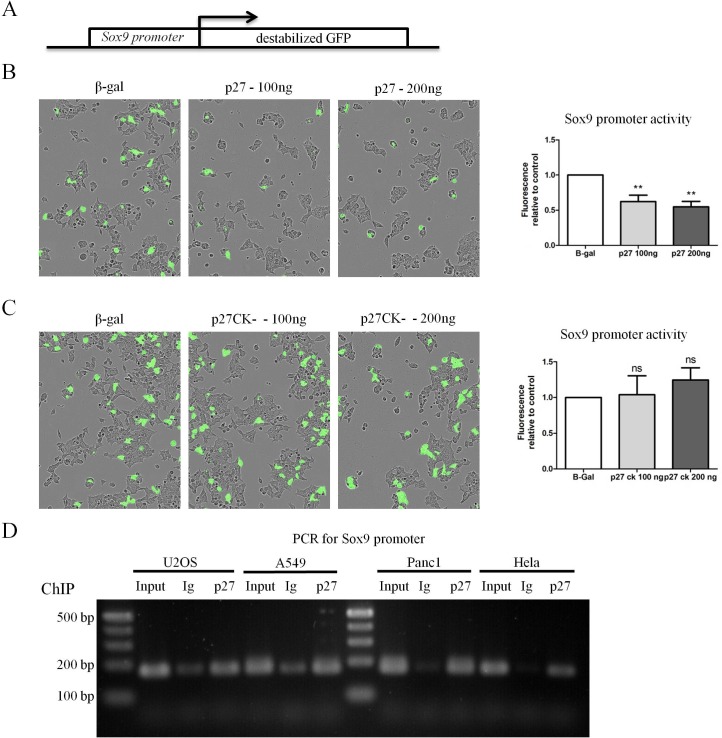
p27 represses transcription of Sox9 in a CDK dependent manner **A.** Schematic of the reporter construct used in transcription reporter assays. The Sox9 promoter is cloned upstream of destabilized GFP (half-life of approximately 1 h). **B.**-**C.** 293 cells were co-transfected with the Sox9 reporter construct or a control reporter construct in which the GAPDH promoter is cloned upstream of GFP and the indicated amount of p27 **B.** or p27CK- **C.** The amount of DNA transfected was normalized using a plasmid encoding β-Gal. Fluorescence levels were monitored and quantified using an Incucyte FLR on 25 images in each well. The graphs in B and C represent the mean fluorescence intensity of the Sox9 promoter normalized to that of the GAPDH promoter in the same condition from six (p27) and five (p27^CK−^) independent experiments. Data were compared by ANOVA followed by Neuman-Keuls multiple comparison test, ** = *p* < 0.01. **D.** A chromatin immunoprecipitation (ChIP) analysis was performed to determine if p27 could bind the Sox9 promoter *in vivo* in various cell lines. PCR products using primers specific for the Sox9 promoter were separated on an agarose gel. For each cell line, PCRs were performed on a fraction of the input and DNA from ChIPs with anti-p27 or isotype control antibodies.

Sox9 and Pdx1 are transcription factors that control an expression program specifying multipotent progenitor cell pools [63]. Their expression can drive the reprogrammation of differentiated acinar cells into primitive ductal progenitors during pancreatitis and in the transformation process leading to PDAC development [51]. p27 was recently found to act as a transcriptional co-repressor by binding to transcription factors such as E2F4/p130 and Ets1 and recruiting HDAC1 and mSin3A to specific promoters [37, 43, 44]. To determine whether p27 may participate in the transcriptional regulation of Sox9 and/or Pdx1 expression, we used transcription reporter assays in which a destabilized GFP cDNA was under the control of the Sox9 or Pdx1 promoter (Figure 4A). Transfection of increasing amounts of p27 decreased Sox9 promoter activity compared to the GAPDH promoter (Figure 4B), indicating that p27 represses the Sox9 promoter.

Consistent with our immunostaining results showing an upregulation of Sox9 in the pancreas of p27^CK−^ mice similar to the p27-null, expression of the p27^CK−^ protein did not repress Sox9 transcription, indicating that the Sox9 promoter is regulated in a CDK-dependent manner by p27 and requires the CDK inhibitory function of p27 (Figure 4C). Therefore, in the context of the regulation of Sox9 expression, p27^CK−^ behaves like a null allele. On the other hand, similar reporter assays conducted on the Pdx1 promoter indicated that p27 does not regulate the activity of the Pdx1 promoter (Supplemental Figure 2), at least not within the sequence used in our experiments corresponding to bp −1187 to −31 from the transcription start site.

Finally, to confirm the direct role of p27 in the control of Sox9 transcription, we performed chromatin immunoprecipitations (ChIP) in various cell lines, including the pancreatic adenocarcinoma cell line PANC1, using p27 antibodies used previously [37, 43, 44] or isotype control antibodies, followed by PCR for the Sox9 promoter sequence. ChIPs with p27 antibodies indicated that p27 is present at the Sox9 promoter, while hardly any signal was detected in the control ChIPs (Figure 4D). In contrast, in similar experiments, p27 was only marginally bound to the Sox2 proximal promoter (−300 to −103 from transcription start site) in these cells (Supplemental Figure 3), consistent with p27 previously reported to regulate Sox2 transcription by binding to the SRR2 enhancer located 4 kb downstream of the Sox2 coding exon [44]. Thus, our results suggest that p27 directly participates in the transcriptional repression of Sox9, but not to that of Pdx1, providing a potential mechanistic explanation for the re-expression of Sox9 in the absence of p27 in the pancreas.

## DISCUSSION

In the nucleus, p27 acts as a tumor suppressor by restraining the activities of cyclin-CDK complexes [3, 4]. Clinical studies supports this role of p27 in the pancreas as nuclear p27 expression is frequently lost in human pancreatic cancer, either by total decrease of protein levels or exclusion from the nucleus, and this correlates with poor prognosis [55-58]. A K-Ras-driven PDAC model in mice also support an important role for p27 in tumor suppression in the pancreas since ablation of one or both p27 alleles considerably decreased survival of the animals [59].

Consistent with previous report indicating that p27 subcellular localization is regulated by Ras activation [8, 17, 19, 34, 35], we found that K-Ras activation in the pancreas caused the cytoplasmic localization, but not degradation, of p27 in pancreatic acinar cells and the different lesions observed during neoplastic progression leading to PDAC formation. Since K-Ras activation is detected in the earliest stage of pancreatic neoplasia [64], p27 mislocalization may also occur very early in humans, as suggested by our analysis of murine pancreata expressing activated K-Ras in the acinar compartment. To determine a potential contribution of p27 mislocalization in these early events leading to tumor formation, we investigated the roles of p27 in the pancreas using two mouse models, either lacking p27 expression, or expressing a mutant form of p27 that lacks only the ability to inhibit CDK complexes but still fulfills its other functions. We found that loss of p27 promoted the mislocalization of acinar polarity markers such as Munc18 and Integrin β1 and the re-expression of ductal progenitor markers like Sox9 and Pdx1. These events have been previously associated with pancreatitis and/or acinar to ductal metaplasia [51, 52, 54, 60-62]. Sox9 expression promotes ADM in mice expressing K-Ras^G12D^ under the control of the Ptf1a promoter and in this model, Sox9 expression was detected in acinar cells, preceding morphological changes induced by ADM and PanNIN formation [51], as we observed in p27 mutant mice. ADM was very rarely observed in pancreata from p27-null mice up to 12 months of age (data not shown), suggesting that loss of p27 (and Sox9 expression) is not sufficient to cause ADM. Thus other events such as K-Ras or EGFR activation may be essential for ADM. Development of pituitary tumors and associated morbidity in p27−/− and p27^CK−^ mice precludes the examination of older animals [5, 30].

Importantly, p27-null and p27^CK−^ pancreas exhibited a similar phenotype (with the exception of Integrin β1 mislocalization), indicating that the loss of p27-mediated CDK inhibition is driving this phenotype. Thus, in the pancreas, the p27^CK−^ mutation essentially behaved like a null mutation. These immunohistological observations were confirmed by the fact that Sox9 transcriptional repression by p27 was also mediated in a CDK-dependent manner as p27^CK−^ expression failed to repress the Sox9 promoter. We cannot exclude that p27 may adopt an oncogenic role in later stages of PDAC development as found in other cells and tissues [19, 30, 31] and it would be interesting to test this possibility by crossing p27^CK−^ mice into an activated K-Ras PDAC model.

We found that p27 repressed Sox9 transcription and associated to the Sox9 promoter in ChIP assays. The Sox9 promoter is regulated by E2F and Ets transcription factors [65-67], it is therefore likely that p27-mediated transcriptional repression of Sox9 is accomplished by recruiting HDAC1 and mSin3a to E2F or Ets-bound promoter, as previously reported [37, 43, 44]. Interestingly, EGFR signaling was recently reported to induce Sox9 transcription in pancreatic acinar cells by the induction of the transcription factor NFATc1 and its association with c-Jun thereby promoting ADM [68]. In our experiments, Pdx1 transcription was not affected by p27 status and an attractive possibility is that Sox9 may promote Pdx1 expression, as reported recently [69].

Overall, the present study suggests that displacement of p27 from the nucleus of pancreatic acinar cells in response to K-Ras activation may constitute an early event promoting metaplasia, notably through the de-repression of Sox9, thereby accelerating tumorigenesis as reported in a p27-null/K-Ras^G12D^ PDAC model [59].

## MATERIAL AND METHODS

### Plasmids and antibodies

Mouse antibodies against β1 integrin (610468), E-cadherin (610182) and Munc18 (610337) were from BD -Transduction Laboratories. Rat anti Laminin α2 (6D580) (sc-71486) and rabbit anti p27 (C-19) (sc-528) were from Santa Cruz Biotechnology. Rabbit anti Sox9 (AB5535) and goat anti Pdx1 (06-1385) were from Millipore. Rabbit anti p27 (RB-9019) was from Thermo Scientific. Secondary antibodies conjugated to Cyanine-2, and -3 were from Jackson ImmunoResearch. The pcDNA3.1+hygro β-Gal (Invitrogen) and pcDNA3.1+hygro p27 and p27CK- have been described previously [38]. Promoter sequences for GAPDH (corresponding to bp −669 to +394 from transcription start site), Sox9 (corresponding to bp −1405 to −153 from transcription start site) and Pdx1 (corresponding to bp −1187 to −31 from transcription start site) were purchased from Origene and cloned into a pZsGreen1-DR vector encoding a destabilized mutant of GFP (Clontech).

### Mice and histology

p27^−/−^ and p27^CK−/CK−^ mice were described previously [5, 8, 30]. p27 mouse lines were maintained in a 129S4 genetic background. ElasK-RasG12V mice, in which the Cre recombinase is expressed under a Tet-off Elastase promoter, allowing K-RasG12V expression in Elastase expressing cells in absence of doxycycline treatment, were described previously [47, 48].

Mice were maintained and procedures performed in accordance with E.U. and national regulations (protocol authorization # 00536.02).

### Immunofluorescence and immunohistochemistry

Dissected pancreas were fixed in 10% formalin overnight, transferred to 70% ethanol for 24 hours, and embedded in paraffin. Paraffin blocks were sectioned at 5μm thickness for histochemistry or immunostaining. Paraffin sections were deparaffinized and either stained with hematoxylin and eosin, or used for immunostaining. Pancreas sections were rehydrated and antigens were unmasked in either sodium citrate (10 mM, pH 6) (Sox9/E-Cadherin staining), low pH (H-3300, Vector Laboratories) (Pdx1/E-Cadherin staining), high pH (H-3301, Vector Laboratories) (p27 RB-9019) or pepsin 0,5% 5mM HCl (Munc18/Lamininα2 and β1-Integrin/Lamininα2 staining) solutions in a steamer for 30 min. Slides were washed twice in PBS-0.2% Triton X-100 and once in PBS. Sections were blocked for 0.5 to 2 h at RT in PBS-0.2% Triton X-100, 10% donkey serum, 3% BSA solution in a humid chamber. Sections were incubated with primary antibodies overnight at 4°C, or 1h at 37°C and washed 3 times in PBS - 0.2% Triton X-100. For immunofluorescence, sections were then incubated for 30 min at 37°C with secondary antibodies conjugated to Cyanine-2, and -3. Slides were washed three times in PBS, and cellular DNA was stained with Hoechst H-33342 at 0.1 μg/mL in the first wash. Slides were mounted with gelvatol (20% glycerol (v/v), 10% polyvinyl alcohol (w/v), 70 mM Tris pH 8). For immunohistochemistry, sections were then incubated for 30 min with Universal ImmPRESS reagent (Vector Laboratories), washed three times 5 min in PBS-0.2% Triton X-100 and staining was visualized using the chromogen 3′3′-diaminobenzidine (ImmPACT DAB, Vector laboratories). Images were captured on a Nikon 90i Eclipse microscope using a CoolSnap HQ camera (Roeper Scientific) or a DS-Fi1 camera (Nikon) and the NIS-Br software (Nikon).

### Cell culture

HEK293T, PANC-1, HeLa, A549 and U-2 OS cells were grown in Dulbecco's modified Eagle medium (DMEM) containing 0.1mM nonessential amino acids, 2 μg/mL penicillin/streptomycin, 4.5 g/L D-glucose, 1 mM sodium pyruvate, and 2 mM L-glutamine (Sigma) and 10% fetal bovine serum (Life Technologies).

### Reporter assays

HEK293T were seeded in 24-wells plates and co-transfected by the calcium-phosphate method for 24 h with 50 ng of either pZsGreen DA (no promoter, negative control), pZsGreen pGAPDH (positive control), pZsGreen pSox9 or pZsGreen pPdx1 and with pcDNA3.1 hygro p27 (0, 100 or 200 ng) or pcDNA3.1 hygro p27CK- (0, 100 or 200 ng). In all conditions, the amount of pcDNA3.1 hygro vector was normalized to 200 ng using the pcDNA3.1 hygro β-Gal plasmid. After 24 h, medium was changed and 12 h later, 25 images per well were captured in phase contrast and fluorescence with an Incucyte FLR automated microscope (Essen BioScience) equipped with a 20x objective. Fluorescence area was quantified on each image using the Incucyte software following manufacturer's recommendations and the values were normalized with the value of pGAPDH fluorescence area in each corresponding condition.

### Chromatin Immunoprecipitations

PANC-1, HeLa, A549 and U-2 OS were grown to confluence. Medium was replaced by PBS and cells were fixed for 10 min by adding Formaldehyde to a final concentration of 1% to cross-link protein-DNA complexes. The cross-linking was stopped by the addition glycine at a final concentration of 0.125 M for 5 min. Cells were rinsed twice with cold PBS and scrapped in cold PBS with Aprotinin, Bestatin, Leupeptin and Pepstatin A at 10 μg/mL. Cells were then pelleted by centrifugation at 3000 rpm for 10 min at 4°C and the pellets were lysed on ice for 30 min using 200 ul/1.10^6^ cells of lysis buffer containing 10 mM Tris-HCl pH 8, 0,25% Triton X-100, 10 mM EDTA, 0,5 mM EGTA, 10 mM Sodium Butyrate, 20 mM β-Glycerophosphate, 100 μM sodium orthovanadate and Protease inhibitor cocktail (Roche). The lysate was then dounce homogenized on ice with 20 strokes to aid in nuclei release and subsequent chromatin shearing. Cells were pelleted by centrifugation at 3000 rpm for 5 min at 4°C, the supernatant was discarded and the pellet containing genomic DNA and cross-linked proteins was then re-suspended in 300 ul/3.10^6^ cells of sonication buffer containing 10 mM Tris-HCl pH 8, 100 mM NaCl, 1 mM EDTA, 0,5 mM EGTA, 10 mM Sodium Butyrate, 20 mM β-Glycerophosphate, 100 μM sodium orthovanadate, 1% SDS and Protease inhibitor cocktail (Roche) and incubated on ice 10 minutes. Chromatin was sheared into fragments between 0.5 kb and 1 kb by sonication for 16 cycles of 30 sec on/20 sec off at 40% amplitude using a Vibra-cell VCX130 Sonicator (Sonics). The sonicated material was clarified by centrifugation at 14000 rpm for 10 min at 4°C. SDS was discarded and supernatant was transferred to new tubes. Chromatin shearing was verified on an aliquot of chromatin decrosslinked by digestion with Proteinase-K and RNAseA on 1% agarose gel. DNA concentration was measured with a NanoDrop spectrophotometer. For each IP, 100μg of sonicated chromatin was used, while 10% of the aliquoted volume was retained for use as input control and stored a −80°C. Sonication buffer was added to the chromatin to a final volume of 800 μl. The Sonication buffer was then converted in RIPA buffer by the addition of 80 μl Triton 10%, 23 μl NaCl 5M and 8 μl Sodium Deoxycholate (DOC) 10%, to each tube. Four μg of antibodies (rabbit anti p27 C19 or rabbit IgG control antibody) and 20 μl of Magna Chip Protein A/G magnetic beads (Millipore) were added per IP and incubated overnight on a rotating wheel at 4°C. Samples were washed 3 times in low salt buffer (10 mM Tris-HCl pH 8, 0,1% Triton X-100, 0.1% SDS, 0.1% DOC, 140 mM NaCl, 1 mM EDTA, 0,5 mM EGTA, 10 mM Sodium Butyrate, 20 mM β-Glycerophosphate and 100 μM sodium orthovanadate), 3 times in high salt buffer (same as low salt buffer with 500mM NaCl), twice with LiCl buffer (0.25 M LiCl, 1% NP-40, 1% DOC, 10 mM Tris-HCl Ph 8, 1 mM EDTA, 1 mM EGTA, 10 mM Sodium Butyrate and 100 μM sodium orthovanadate) and finally twice with Tris-EDTA buffer (TE). Subsequent elution and purification of the immunoprecipitated DNA-proteins complexes was performed with an IPure kit (Diagenode) according to manufacturer's instructions. PCR for the Sox9 promoter were performed using 20 ng of template DNA with Phusion HotStart II DNA polymerase (Thermo Scientific) for 35 cycles (denaturation 98°C for 10 sec; annealing 60°C for 10sec; elongation 72°C for 15 sec). The primer sequences were: SOX9_F: 5′-GCGGAGAGAGCAGTGAAAAG-3′; SOX9_R: 5′-CCGGGACTTCCAAGTGTGTA-3′. The 165 bp PCR product corresponds to nucleotides −406 to −241 from the human Sox9 transcription start site.

### Statistical analysis

All statistical analyses were performed using Graphpad Prism 5.0 software. Differences between groups were considered significant when P < 0.05. All data were compared by a one-way analysis of variance (ANOVA) followed by Neuman-Keuls multiple comparison tests.

## SUPPLEMENTARY MATERIAL FIGURES


