# Supplementary material for: Loss of p27Kip1 promotes metaplasia in the pancreas via the regulation of Sox9 expression
Source: Oncotarget. 2015 Sep 21;6(34):35880–92. doi: 10.18632/oncotarget.5770 (PMC4742148; doi:10.18632/oncotarget.5770)
Supplement: Supplementary file 1 [file oncotarget-06-35880-s001.pdf]

Loss of p27[Kip1] promotes metaplasia in the pancreas *via* the regulation of Sox9 expression

Supplementary Material

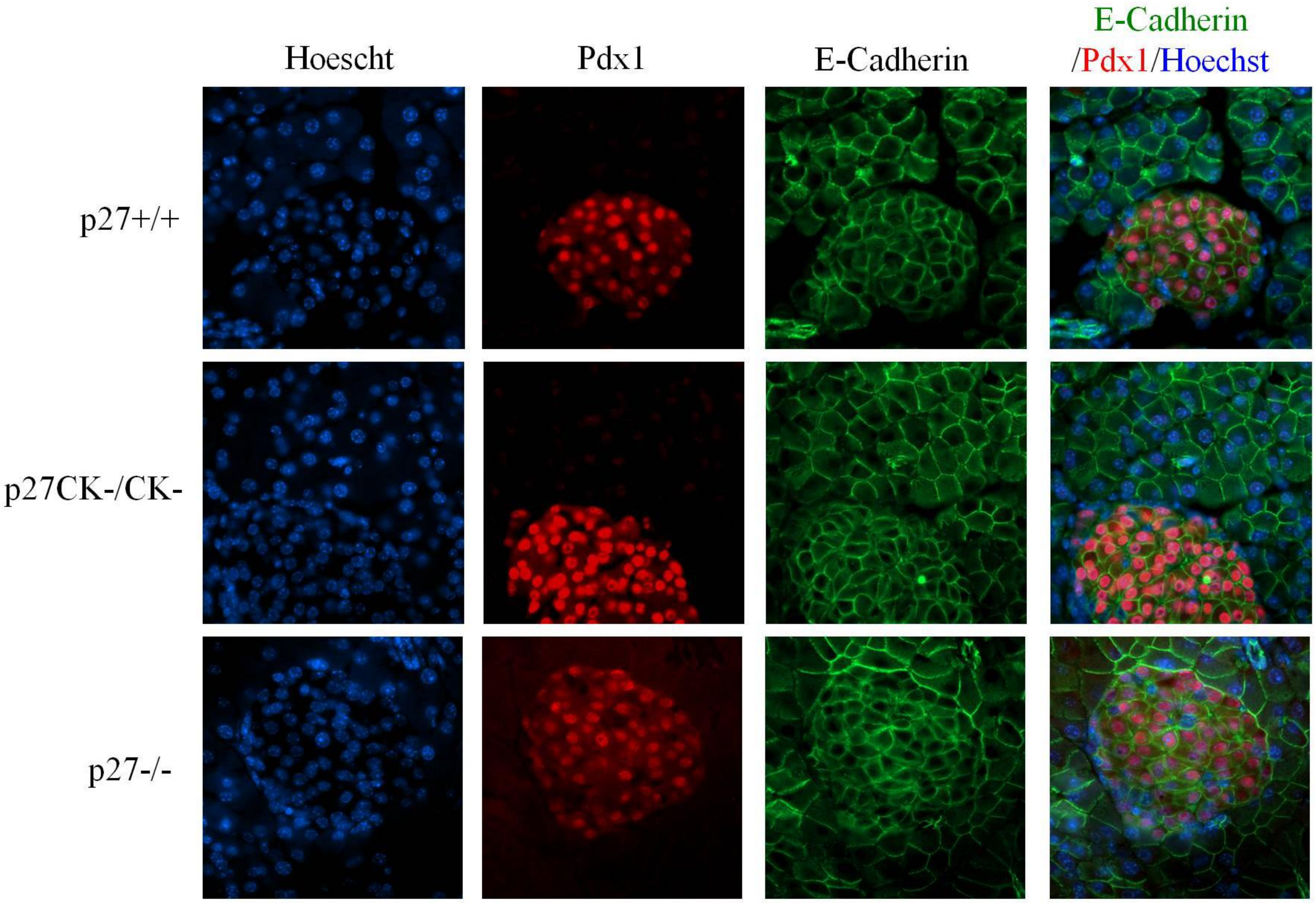

Supplementary Figure 1 : Expression of Pdx1 in islets of Langherans. Paraffin-embedded pancreas sections from p27+/+, p27-/-, and p27CK-/CK- mice were stained with E-Cadherin and Pdx1 antibodies. DNA was stained with Hoechst. All images were acquired using a 60x objective.

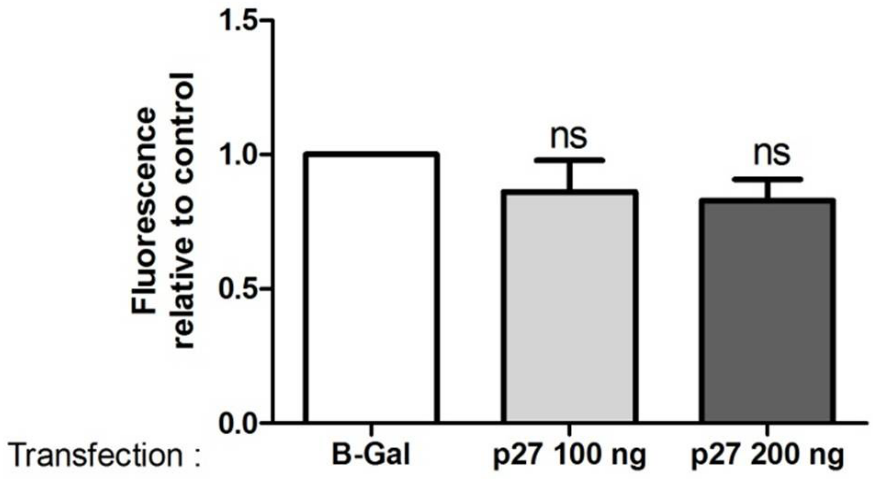

Supplementary Figure 2 : p27 does not regulate transcription of Pdx1. Reporter assays were realized in HEK 293 cells co-transfected with a reporter construct expressing destabilized GFP under the control of the Pdx1 promoter and a plasmid encoding p27. A plasmid encoding β-Gal was used to normalize the amount of transfected DNA in all conditions. Fluorescence levels were monitored and quantified using an Incucyte FLR (EssenBioscience). The graph shows the mean fluorescence area of the Pdx1 promoter normalized to that of the GAPDH promoter in the same condition from five independent experiments. Data were compared by ANOVA followed by Neuman-Keuls multiple comparison test.

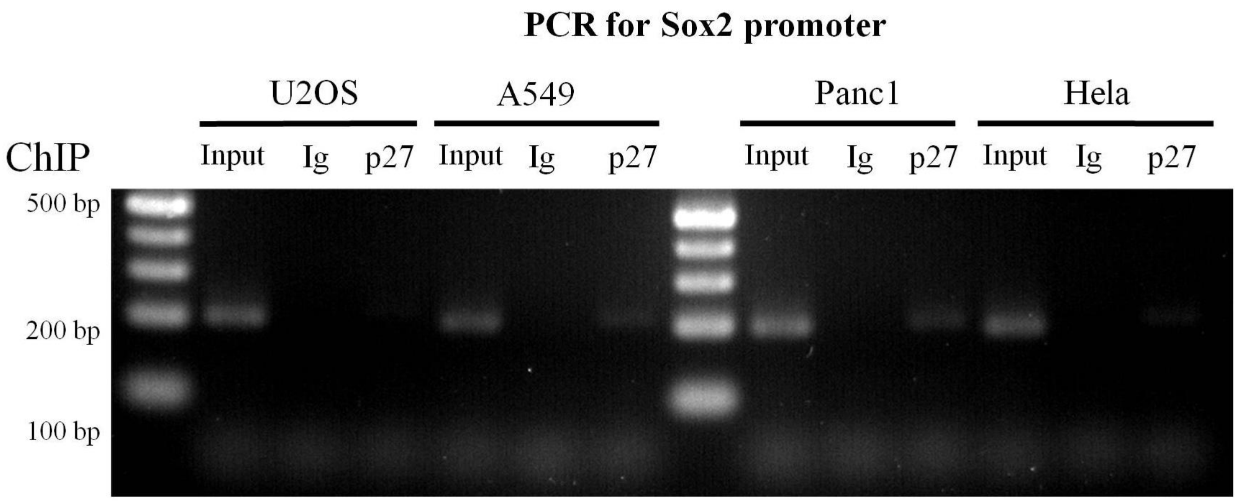

Supplemental Figure 3: Chromatin immunoprecipitation (ChIP) analysis was performed to determine if p27 could bind the Sox2 promoter *in vivo* in various cell lines. For each cell line, PCRs were performed on a fraction of the input and DNA from ChIPs with anti-p27 (polyclonal C-19) or isotype control antibodies. PCR products using primers specific for the Sox2 promoter were separated on an agarose gel. PCR conditions are described in the methods section and the primers used were: SOX2-ChIP-F5 5'-TTTGCAAAAGGGGGAAAGTA-3' and SOX2-ChIP-R5 5'-GAGGCAAACTGGAATCAGGA-3'. The 198 bp PCR product corresponds to nucleotides -300 to -103 from the human Sox2 transcription start site.
